# Construct and Criterion Validity of the Euro Qol-5D in Patients with Systemic Lupus Erythematosus

**DOI:** 10.1371/journal.pone.0098883

**Published:** 2014-06-03

**Authors:** Su-li Wang, Bin Wu, Li-an Zhu, Lin Leng, Richard Bucala, Liang-jing Lu

**Affiliations:** 1 Department of Rheumatology, Ren Ji Hospital, School of Medicine, Shanghai Jiao Tong University, Shanghai, China; 2 Clinical Outcomes and Economics Group, Department of pharmacy, Ren Ji Hospital, School of Medicine, Shanghai Jiao Tong University, Shanghai, China; 3 Department of Medicine, Section of Rheumatology, Yale University School of Medicine, The Anlyan Center, New Haven, Connecticut, United States of America; Keio University School of Medicine, Japan

## Abstract

**Objective:**

To investigate the construct and criterion validity of the Euro Qol-5D (EQ-5D), which allows quality-adjusted life-years to be calculated, in patients with systemic lupus erythematosus (SLE).

**Methods:**

Consecutive SLE patients who had been followed at the Renji Hospital, School of Medicine, Shanghai Jiao Tong University were recruited. Cross-sectional correlations of the EQ-5D with equivalent domains in disease-specific health-related quality of life (HRQoL), LupusQol, Systemic Lupus Erythematosus Disease Activity Index (SLEDAI) measures, the Systemic Lupus International Collaborating Clinics Damage Index (SDI), and patient characteristics were tested. Discriminant validity to assess the ability to distinguish between patients of different disease severity was assessed. There also were evaluations of ceiling and floor effects.

**Results:**

240 patients were recruited in total. The EQ-5D correlated moderately to strongly with all domains of the LupusQoL (r: 0.44–0.7) apart from intimate relationships (r = 0.25) and body image (r = 0.18). There was moderate negative correlation between EQ-5D and clinical assessment of disease, SLEDAI (r = −0.589) and SDI (r = −0.509). When compared with equivalent domains on LupusQoL, there was good construct validity in EQ-5D (r: 0.631–0.812). EQ-5D could also discriminate patients with varied disease severity (according SLEDAI and SDI). There was no floor effect in EQ-5D but the ceiling effect remains strong (34%).

**Conclusion:**

Our results provide sufficient evidence that the EQ-5D displays construct and criterion validity for use in SLE patients. Disease-specific measures of HRQoL used alongside may be a better choice.

## Introduction

Systemic lupus erythematosus (SLE) is a chronic inflammatory autoimmune disease characterised by the deposition of immune complexes in various tissues, which is found mainly in women during the childbearing years and is particularly common in Asian, and African American/Caribbean individuals [1]–[3]. Despite little conspicuous progress in the treatment of SLE, long-term survival has significantly improved [3]. At present, the health related quality of life (HRQoL) of patients with SLE is under increasing attention [4] as the HRQoL among SLE patients is worse than the general population, even compared with other rheumatic diseases [5]. As might be expected, a series of novel therapies are being developed for SLE [6]. For example, belimumab, a B cell modulator, is the first to demonstrate success in phase III studies and has received marketing authorization [7]. However, before widespread use in clinics, new therapies require evaluation for cost utility, which is of vital concern to policy makers.

Cost-utility analysis requiring quality adjusted life years (QALY) to measure the unit of health-gain is the most commonly used method [8] and compares interventions in terms of their cost per unit of effect. Where two or more interventions are found to achieve the same level of benefits, the one with the least cost is considered the most cost-effective alternative [8]. Generic preference-based measures, such as EQ-5D, SF-6D and HUI, have become widely used in economic evaluation, and have gained popularity to obtain health state value to calculate QALY over the last decade [9]. This development has arisen in part from their ease of use and their alleged generic properties. Assessment of HRQoL in patients with SLE also can be provided by disease-specific measures such as LupusQoL [10]; as they are designed for SLE, the results of these may be more specific. However, these disease-specific measures do not provide a single value for cost-utility analysis, which is the concern of the policy makers.

EQ-5D is a generic preference-based measure of health developed by a multidisciplinary group of researchers [11], [12]. It has a structured health state descriptive system with five dimensions: mobility, self-care, usual activities, pain/discomfort and anxiety/depression. There are two types depending on the number of levels in each domain. The one with five levels, which was considered more friendly to users, was used in the present study. These five dimensions together define a total 5^5^ health states formed by different combinations of levels. As a simple instrument, EQ-5D is widely used in various diseases. But the validity in SLE is not well established, especially in Chinese SLE patients. In this study, we collected and analyzed clinical data, HRQoL data, and socioeconomic data to examine the construct validity of the EQ-5D in patients with SLE.

## Patients and Methods

This study was approved by the Institutional Review Board of Shanghai Jiao Tong University and the Ethics Committee of Renji Hospital. These committees specifically approved that written informed consent was not required because data were going to be analysed anonymously. Following feedback from participants in the pilot study, all participants granted oral consent after receiving comprehensive information about the study. Oral consent was documented by interviewers at the beginning of the patient interview.

### Patients

Consecutive SLE patients were included, who were followed at the Renji Hospital, School of Medicine, Shanghai Jiao Tong University from March 2012 to May 2013. All patients fulfilled the 1997 revised American College of Rheumatology classification criteria for SLE [13], and had received stable therapy for at least 2 months.

### Data collection

At baseline all patients underwent a clinical interview and examination to collect demographic information, including age, disease duration, age at protocol entry, clinical manifestations at disease onset, cumulative clinical manifestations, education and marital status.

The clinical assessment included evaluation of disease activity using the Systemic Lupus Erythematosus Disease Activity Index(SLEDAI) [14] and cumulative damage using the Systemic Lupus International Collaborating Clinics (SLICC)/ACR Damage Index (SDI) [15]. The SLEDAI is a 24-item instrument for assessing SLE activity in nine organ systems, each item with a weighting from 1 to 8 depending on severity; the score ranges from 0 (no activity) to 105 (maximum activity) [14]. Clinical and laboratory data are required to complete the questionnaire. SLICC/ACR-DI (SDI) reports disease damage based on the evaluation of 12 organ systems. The dysfunction must be present for 6 consecutive months. The score ranges from 0 (no damage) to 46 (maximum damage), with higher scores signifying more damage.

All patients completed the generic preference-based measurement of health, EQ-5D-5L at baseline, each domain of which had 5 levels: no problems, slight problems, moderate problems, severe problems, and extreme problems. Scores for the five domains in EQ-5D were generated. Scoring algorithm estimated from the valuation survey undertaken by the UK Measurement and the Valuation of Health (MVH) group was used because of its widest popularity. The best possible score on the EQ-5D is 1 (equivalent to full health) and the worst possible score is −0.594 (presenting a state worse than death).

LupusQoL, a lupus-specific HRQoL questionnaire, which had been modified for applicability to Chinese SLE patients [16], also was completed. It consists of 34 items grouped in eight domains: physical health (PH), pain (PN), planning (PL), intimate relationships (IR), burden to others (BU), emotional health (EH), body image (BI) and fatigue (F) and has a five-point Likert response format, where 4 = never, 3 = occasionally, 2 = a good bit of the time, 1 = most of the time, and 0 = all of the time [10]. LupusQoL is scored for each domain as the mean domain score; the transformed scores range from 0 (worst) to 100 (best).

We used both disease activity and damage to define disease severity of SLE, which were determined by SLEDAI and SLICC-DI [16].

### Statistical Analyses

The data were analyzed cross-sectionally at baseline.

Convergent validity and discriminant validity were used to assess the construct validity of EQ-5D, which reflected the sensitivity and specificity of the measure. Convergent validity was assessed by measuring the extent of correlation of EQ-5D with the domains of the LupusQoL, SLEDAI (for activity), SDI (for damage), and characteristics of patients (age, disease duration and education). The extent of correlation between observed relationships of the concepts and the hypothesized concepts also were measured to assess the convergent validity. A strong correlation was defined as≥0.70, moderate to substantial as 0.30–0.70, and weak as <0.30 [17]. We expected that there would be moderate to strong correlations between EQ-5D and LupusQoL because the latter might be the closest measure to the gold standard of HRQoL in SLE patients [18]. Discriminant validity was used to assess whether the instrument could distinguish between patients of different disease severity. Patients were divided into two groups by a SLEDAI score cutoff of 4 or SLICC-DI score cutoff of 1 [19]. It was hypothesized that LupusQoL domains would be significantly altered in these two groups and an ordinary least-squares regression was used to test this possibility [18]. Effect sizes (Cohen's D) were calculated to quantify the magnitude of the differences in SD units by dividing the mean difference in EQ-5D by the standard deviation for both groups combined [18]. It was suggested that an effect size of 0.2 is small, 0.5 is moderate, and 0.7 is large [18].

Floor and ceiling effects were examined to explore potential to detect change. Ceiling effect exists if a large number of respondents occupy the best possible health state of a measure; a floor effect is just the opposite. If a ceiling effect exists, the ability of the measure to detect any further better states of health is inhibited and floor effect limits the ability to detect further worsening [20], [21]. A ceiling/floor effect is considered to exist when >15% of respondents fall into the ceiling/floor [21]. The 5 domains of the EQ-5D with 5 levels and the overall score of EQ-5D were tested for ceiling or floor effects. When floor/ceiling effects were found to be serious, comparisons were made with responses to similar domains of LupusQoL.

We used SPSS software, version 10.0 to analyze data. Descriptive statistics were reported. The continuous variables were tested for normality; a non-parametric test (Mann-Whitney) was used for comparing continuous data.

## Results

Among the 240 patients who participated in this study, complete data were available for 214 patients. 201(93.9%) patients were women; all are Chinese. At baseline the mean (SD) age and disease duration were 33.8 years (±9.2) and 4.8 years (±4.4), respectively. The mean (SD) SLEDAI and SDI were 2.9 (±3.9) (median 2, range 0–25) and 0.36(±0.9) (median 0, range 0–6).

### Construct and criterion validity

There were positive correlations between EQ-5D score and all domains of LupusQoL (Table 1). The correlations were moderate to strong (r = 0.4–0.8) for all domains of LupusQoL except intimate relationships (r = 0.252) and body image (r = 0.179), which were weakly correlated to EQ-5D score. The correlations of the EQ-5D with the disease-specific measures were moderate for the SLEDAI score (r = −0.589) and SDI (r = −0.509) in the expected direction. There were no correlations between EQ-5D and patient characteristics such as age (r = −0.141) disease duration (r = −0.104) and education (r = 0.238).

**Table 1:** Correlations of EQ-5D with SLE measures/patient characteristics (correlations are Spearman unless specified).

	Correlation R	P
LupusQoL		
Physical health	0.603	p<0.01
Pain	0.703	p<0.01
Planning	0.45	p<0.01
Intimate relationships	0.252	p<0.01
Burden to others	0.437	p<0.01
Emotional health	0.576	p<0.01
Body image	0.179	p<0.01
Fatigue	0.544	p<0.01
Disease activity/damage		
SLEDAI	−0.589	p<0.01
SDI	−0.509	p<0.01
Patient characteristics		
Age*	−0.141	p>0.01
Disease duration	−0.104	p>0.01
Education	0.238	p>0.01

SLEDAI: SLE Disease Activity Index; SDI: Systemic Lupus Collaborating Clinics Damage Index.

*Pearson correlation.

The EQ-5D domains had good construct validity when compared with equivalent domains of LupusQoL (Table 2).

**Table 2:** Convergent validity of EQ-5D used in SLE patients.

EQ-5D domains	LupusQoL domains	Spearman's r
Self-care	Physical health	0.631
Usual activity	Physical health	0.747
Pain/Discomfort	Pain	0.812
Anxiety/Depression	Emotional health	0.767

### Discriminant validity

The mean EQ-5D scores of patients with high disease activity (SLEDAI>4) was lower than those with low disease activity (0.619 vs. 0.846; EQ-5D: B coefficient −0.028, p<0.01; Table 3). Similarly, it was lower in patients with damage associated with SLE (SDI>1) (0.663vs.0.843; EQ-5D: B coefficient −0.107, p<0.01; Table 3). The effect size (ES) of the difference in means suggested that the differences were moderate for SDI (ES = 0.697) and large for SLEDAI (ES = 0.941). It's suggested that EQ-5D could discriminate subjects with different disease severity, which was associated with different health states.

**Table 3:** Discriminant validity of EQ-5D used in SLE patients with disease activity and damage as the external anchors.

	EQ-5D, mean(SD)	P	Effect Size
Disease activity		<0.01	0.941
SLEDAI≤4	0.846(0.134)		
SLEDAI>4	0.619(0.261)		
Damage		<0.01	0.697
SDI≤1	0.843(0.137)		
SDI>1	0.663(0.260)		

SLEDAI: SLE Disease Activity Index; SDI: Systemic Lupus Collaborating Clinics Damage Index.

Ordinary least-squares regression was used.

### Ceiling effect and floor effect

There were no floor effects for the preference-based score and domains of EQ-5D. But serious ceiling effects were found to exist for both EQ-5D preference-based score and domain scores (34%; 22.8–47.9%), especially in the self-care domain. Almost half of the individuals (47.9%) responded with the ideal response “no problems” (Table 4). In the comparable domains of LupusQoL, patients at the ceiling of self-care, usual activity, pain/discomfort in EQ-5D also had a high median LupusQoL physical health score (90, IQR 81, 97) and pain score (92, IQR 75, 100).

**Table 4:** Ceiling effect and floor effect of EQ-5D used in SLE patients.

EQ-5D Subscale level	mobility	self-care	usual activities	pain/discomfort	anxiety/depression	EQ-5D Score	
1	30.8	47.9	45.2	47.2	22.8	1	34
2	42.1	25.1	25	36.9	41.3	−0.594	0
3	23.3	14.7	15.1	10.7	24.7		
4	2.8	11.9	13.7	4.2	7.5		
5	0.9	0.5	0.9	0.9	3.7		

## Discussion

Data from studies assessing the HRQoL of SLE patients have shown that even with inactive disease, patients with SLE had a poorer HRQoL when compared to healthy subjects [22], [23]. With patients enjoying a longer life span, interest in the HRQoL of SLE patients has gained growing attention. As the simplest generic preference-based measure, EQ-5D is widely used to make cost-utility analysis in various diseases, which is critical to policy-makers in health economics [24].

Our study provides evidence that EQ-5D is a valid measure for use in SLE. Because studies in China that directly elicit preferences from general population samples to derive value sets for the EQ-5D-5L are still under development, we used the UK value set in our study [12].

The present results include validity of EQ-5D against another well validated-tool LupusQoL. All domains have moderate to strong correlations with score of EQ-5D, except body image and intimate relationships, which are important aspects of HRQoL in SLE patients. This result reinforces the need to collect disease-specific measures of HRQoL alongside generic preference-based instruments. It also was found that the EQ-5D was differentiated between patients with different disease severity; this suggests it has the ability to distinguish patients with different health status, which plays an important role in clinical practice.

Serious ceiling effects are observed, especially in self-care and pain/discomfort, although EQ-5D-5L was established to reduce ceiling effects. This result indicates that health status above the highest level the instrument can measure is not accurately estimated. The health status distribution of patients in the study also should be taken into account. As an additional limitation, outpatients with inactive disease that were recruited into our study comprised a significant percentage and individuals with relatively better health status might also consist of a majority. Moreover, the high median score of the comparable domains of LupusQoL may also reflect these possibilities. Perhaps this limitation further contributed to the observed ceiling effect.

There is another limitation that must be considered: the population in our study included only Chinese patients. We will need to examine this scale in a more diverse population and include subjects from other ethnic backgrounds in order to understand it more comprehensively.

## Conclusions

Sufficient data are available to indicate that the reliability and validity of EQ-5D among patients with SLE are acceptable. Disease-specific measures of HRQoL used alongside generic preference-based instruments are necessary to evaluate the actual health status. Further work remains to be done, including confirming its applicability in multi-ethnic SLE populations and exploring its precise value for clinical practice.
